# Profile and Treatment Outcomes of Tuberculosis in the Elderly in Southeastern Nigeria, 2011–2012

**DOI:** 10.1371/journal.pone.0111910

**Published:** 2014-11-04

**Authors:** Daniel C. Oshi, Sarah N. Oshi, Isaac Alobu, Kingsley N. Ukwaja

**Affiliations:** 1 Centre for Development and Reproductive Health, Enugu, Enugu State, Nigeria; 2 National Tuberculosis and Leprosy Control Programme, Ministry of Health, Abakaliki, Ebonyi State, Nigeria; 3 Department of Internal Medicine, Federal Teaching Hospital, Abakaliki, Ebonyi State, Nigeria; St. Petersburg Pasteur Institute, Russian Federation

## Abstract

**Background:**

The demographic transition and increasing life expectancy in Africa has lead to a rising elderly population. In Nigeria, little is known about the profile of and treatment outcomes of tuberculosis (TB) in the elderly.

**Methods:**

Retrospective cohort study of adult TB patients treated between January 2011 and December 2012 in two large health facilities in Nigeria. The demographic, clinical and treatment outcomes of patients aged 60 and older were compared with those aged 15 to 59 years.

**Results:**

Elderly (≥60 years) TB patients accounted for 12.7% of all (1668) adult TB enrolled. Elderly patients had a higher proportion of men compared to non-elderly (64.2% vs 56.8%; p = 0.043); but a lower proportion of smear-positive TB at baseline (40.7% vs 65.8%; p<0.001). A higher proportion of elderly patients failed to smear convert after the intensive phase of treatment (23.7% vs 19.8%; p = 0.06), and overall elderly patients had lower treatment success rates (68.9% vs 77.1%; p = 0.009). Unsuccessful outcomes were mainly due to higher default and deaths in the elderly. The risk factors for unsuccessful outcomes in the elderly were: extrapulmonary TB case (adjusted odds ratio (aOR) 10.9; 95% confidence interval (CI) 1.1–108), and HIV co-infection (aOR 3.6; CI 1.1–11.7).

**Conclusions:**

Treatment outcomes of elderly TB patients were inferior to non-elderly adults with higher death and default rates being implicated. With the rising elderly population, specific strategies are needed to quickly address TB management in the elderly in resource-limited settings.

## Introduction

Globally, the frequency of tuberculosis among elderly persons is about three times that observed in non-elderly adults [1]. The increased risk of tuberculosis in persons aged 60 years and above is being driven by immunosuppression, malnutrition, poverty, reduced access to health services, and co-morbidities like diabetes mellitus [2]–[3]. Also, with age-related physiological, psychological, physical disability and social changes compounded by chronic degenerative diseases, TB in elderly (≥60 years) individuals is likely to follow a non-classical course [4]. Previous studies have demonstrated that besides cough, other classical features of pulmonary TB like fever, night sweats, and hemoptysis have lower rates in elderly patients [5]. Moreover, tuberculosis treatment in elderly individuals have been found to be complicated by treatment for co-morbid diseases leading to a consequent increase in adverse drug effects, mortality, treatment default and increased rates of retreatment and drug resistance [6]–[8].

With the current demographic transition and increasing life expectancy in low- and middle-income countries [9], the proportion of elderly persons is increasing, and the incidence of TB among them is expected to increase. Thus, failure of early identification and management of TB in the elderly can present major challenges for a TB control programme as affected elderly patients may have increased morbidity and mortality and they can become an important source of infection – perpetuating the chain of transmission in the community [10]–[12]. Moreover, age-related factors have been found to enhance the susceptibility to TB infection, abetting outbreaks in institutionalized settings [12]–[13]. To date, several published studies have reported on TB and its treatment outcomes in the elderly in India, Taiwan, Hong Kong, U.S.A, Mexico, Brazil and Germany [3]–[4], [10]–[12], [14]–[18]. Data from African populations have been scarce [19]. To optimize healthcare services and TB control in resource-poor settings of Africa, detailed knowledge of the epidemiological features of TB and its treatment outcomes in the elderly in the region are needed.

The aim of this study was to describe the epidemiological characteristics and treatment outcomes of elderly patients with tuberculosis in a high-incidence African setting where patients were managed within a TB control programme with significant resource limitations. Specific objectives were to determine among adult TB patients in Nigeria in 2011 and 2012: (i) the proportion of elderly (≥60 years) persons with TB and compare them with non-elderly adult TB cases in terms of their demographic and clinical characteristics, (ii) the differences in the treatment outcomes of elderly and non-elderly TB cases stratified by: gender, type of TB, TB regimen received, residence, treatment category, HIV status and facility where care was given, and (iii) the determinants of unsuccessful treatment outcomes among elderly TB patients.

## Materials and Methods

### Ethical statement

The study complies with international guidelines for research and was approved by the Ethics and Research Advisory Committee of the National Tuberculosis Control Programme, Ministry of Health, Ebonyi State, Nigeria. As this was a retrospective study, the consent of the patients was not obtained; however, patient records was anonymized and de-identified prior to analysis.

### Study design

This was a retrospective cohort study using routine programme data from two hospitals in the Nigerian state of Ebonyi. It included all elderly and non-elderly adult TB patients registered for treatment between 1 January 2011 and 31 December 2012.

### Study setting

The study was conducted in one rural faith-based (private) secondary care hospital and the only tertiary (public) hospital in Ebonyi State, Southeastern Nigeria. Ebonyi state is one of Nigeria's 36 states; it has an estimated population of 2.5 million people [20] and 13 local government areas – with 130 health care facilities registered with the Nigeria National Tuberculosis and Leprosy Control Programme (NTP) as TB care providers. Also, the state of Ebonyi notifies about 2300 TB patients annually [21]. The study hospitals were selected due to high TB notification rates and to allow for public–private mix comparison. Together the two hospitals notified about 50% of all TB cases in Ebonyi state in 2009 [21], and they serve an estimated 1.5 million people in the state.

### TB diagnosis, treatment and follow-up

Persons who developed a cough lasting two or more weeks with or without night sweats, fever, weight loss, shortness of breath, and sputum production were evaluated, registered and treated according to the WHO/NTP guidelines [22]–[23]. TB patients are treated using the community-based directly observed treatment short course (DOTS) strategy. Briefly, new TB patients were treated daily either using the 8-month anti-TB regimen (in 2011) or the 6-month regimen (in 2012). The 8-month regimen comprised of a two-month intensive phase using rifampicin (R), isoniazid (H), pyrazinamide (Z) and ethambutol (E) and a six-month continuation phase comprising of ethambutol and isoniazid (2RHZE/6EH). The 6-month regimen was adopted by the NTP in 2012 in line with the new WHO guideline [23]. It consisted of two months of RHZE and four months of RH (2RHZE/4RH). Retreatment patients are given a three month intensive phase comprising of RHZE with streptomycin in the first two months, followed by RHZE for one month; and a continuation phase of rifampicin, isoniazid and ethambutol for five months. The community-DOTS approach requires that a family member/community volunteer (DOTS-supporter) monitors the daily intake of the medications by the patients.

Treatment follow-up was done using clinical and laboratory evaluations at intervals according to standard methods [22]–[23]. Clinical follow-up involved regular clinical assessments including weight monitoring and assessment of patients' drug intake records for regularity during drug collection visits. Laboratory follow-up involved sputum microscopy for acid-fast bacilli at specified intervals. For smear positive TB patients, two sputum samples were collected and examined at the end of 2^nd^ month of treatment for new cases or 3^rd^ month of treatment for re-treatment cases (if any of these sample tests positive for acid-fast bacilli, the intensive treatment phase was extended by one-month and smear examination repeated at the end of 3rd month (new cases) or 4th month (re-treatment cases) of treatment before commencing the continuation phase) [22]–[23]. Also, another sputum examination was done at the end of 5th month of treatment as well as at the end of 6th month for (six month regimen) or end of 7th month (for eight month regimen) of treatment. For smear negative patients, sputum samples were examined only at the end of the 2nd month of treatment [22]–[23]. A TB patient who missed a scheduled appointment/drug collection visit is contacted through phone calls to resume treatment; while a patient lost-to-follow-up is visited by a community health worker.

### Population

The study included all elderly TB patients (≥60 years) and non-elderly adult TB patients (15–59 years) registered for treatment at the two study hospitals between 1 January 2011 and 31 December 2012.

### TB treatment outcomes

Treatment outcomes were defined in accordance with standard WHO and NTP definitions as follows [22]–[23]: cured (a patient who was initially smear-positive and who was smear-negative in the last month of treatment and on at least one previous occasion), completed treatment (a patient who completed treatment, but who did not meet the criteria for cure or failure - this definition applies to smear-positive and smear-negative patients and to patients with extrapulmonary TB), died (a patient who died regardless of the cause during treatment), treatment failure (a patient who was initially smear-positive and who remained smear positive at month 5 of treatment or later during treatment), lost to follow-up (a patient whose treatment was interrupted for 2 consecutive months or longer), and transferred-out (a patient who transferred to another reporting unit and for whom treatment outcome is unknown). A patient whose final outcome was either cured or completed treatment had a “successful outcome” while any other outcome was classified as “unsuccessful”.

### Data collection

Data were sourced from the TB treatment registers of the study sites, were checked for errors, double-entered, and analyzed using Epi Info 3.4.1 (CDC, Atlanta, GA USA). The following variables were collected: TB registration number, age, gender (female vs. male), treatment category (new vs. retreatment case), residence (rural vs. urban), type of TB (pulmonary vs. extrapulmonary TB), human immunodeficiency virus (HIV) status (HIV-positive vs. HIV-negative), health facility where care was given (private vs. public) treatment regimen received (6-month vs. 8-month regimen), sputum positivity before starting treatment and at the end of the intensive phase, and treatment outcomes.

### Statistical analysis

Categorical group comparisons between elderly and non-elderly patients were made using the Chi-square test. Relative differences in rates of unsuccessful outcomes between these two groups were compared using crude odds ratios (ORs) and adjusted ORs. Adjusted ORs were determined through multivariable logistic regression analysis. We performed a stratified analysis to determine the occurrence of interaction and confounding between the outcome variable and the explanatory variables. All variables that were of clinical importance and all with a univariate *P*<0.25 were included in the multivariable model. A backward elimination approach was used to find the best model. *P*<0.05 were considered statistically significant.

## Results

### Characteristics of the study population

Of 1668 TB patients comprising of 1574 pulmonary TB (985 smear-positive and 589 smear-negative cases) and 94 extrapulmonary TB enrolled; 212 (12.7%) were elderly (≥60 years) and 1456 (87.3%) were non-elderly. The mean (SD) age of the elderly patients was 66.7 (6.5) years. Demographic and clinical characteristics of elderly and non-elderly patients are shown in Table 1. Elderly patients had a higher proportion of men compared to non-elderly (64.2% vs 56.8%; p = 0.043). A higher proportion of the elderly resided in a rural area (79.2% vs 69.5% among non-elderly; p = 0.003); but, a lower proportion of them were HIV-positive (7.1% vs 22.5%; p<0.001). There were no differences in the proportion of elderly and non-elderly patients in terms of facility where care was given, treatment category, regimen received and type of TB (Table 1). However, among elderly pulmonary TB patients, there was a lower proportion of smear-positive TB at baseline 83 (40.7%) vs 902 (65.8%) among non-elderly (p<0.001).

**Table 1:** Demographic and clinical profile of elderly versus non-elderly TB patients, Nigeria, 2011–2012.

Variables	Non-elderly n (%)	Elderly (≥60) n (%)	*P* - value
Total	1456 (87.3)	212 (12.7)	
Gender			0.043
Female	629 (43.2)	76 (35.8)	
Male	827 (56.8)	136 (64.2)	
Residence			0.003
Rural	1012 (69.5)	168 (79.2)	
Urban	444 (30.5)	44 (20.8)	
Facility			0.65
Private	1211 (83.2)	179 (84.4)	
Public	245 (16.8)	33 (15.6)	
Treatment category			0.61
New	1353 (92.9)	199 (93.9)	
Retreatment	103 (7.1)	13 (6.1)	
Type of TB			0.21
Pulmonary TB	1370 (94.1)	204 (96.2)	
Extrapulmonary TB	86 (5.9)	8 (3.8)	
HIV status			<0.001
HIV – positive	327 (22.5)	15 (7.1)	
HIV – negative	1129 (77.5)	197 (92.9)	
Treatment Regimen			0.13
Regimen 1	757 (52.0)	122 (57.5)	
Regimen 2	699 (48.0)	90 (42.5)	

Regimen 1 = 8-month regimen; Regimen 2 = 6-month regimen; TB = tuberculosis; HIV = human immunodeficiency virus.

### Treatment outcomes

Table 2 shows the treatment outcomes for elderly and non-elderly patients according to the type and category of TB. Among all TB cases, elderly patients had lower rates of successful outcomes (68.9%% vs 77.1%; p = 0.009). The proportion of death and loss to follow-up were higher among the elderly (12.3% vs 9.5% (p = 0.1) and 12.3% vs 9% (p = 0.07) respectively, but the differences did not reach statistical significance. No difference was also observed with rates of treatment failure. Except for retreatment TB cases, among pulmonary TB, extrapulmonary TB, and new TB cases, treatment success rates were significantly higher in non-elderly compared to elderly patients (Table 2). Also, the proportion of elderly outnumbers non-elderly patients in all the unfavorable outcomes like death, treatment failure, and default although most of these differences did not reach statistical significance (Table 2).

**Table 2:** Treatment outcomes stratified by type of TB and treatment category in Nigeria, 2011–2012.

Treatment outcome	Non-elderly n (%)	Elderly n (%)	*P*-value
**All TB Cases**			0.009
Successful	1122 (77.1)	146 (68.9)	
Unsuccessful	334 (22.9)	66 (31.1)	
Failure	23 (1.6)	3 (1.4)	0.9
Death	139 (9.5)	26 (12.3)	0.1
Default	131 (9.0)	26 (12.3)	0.07
Transfer-out	41 (2.8)	11 (5.2)	0.04
Total			
**Pulmonary TB**			0.01
Successful	1080 (78.8)	145 (71.1)	
Unsuccessful	290 (21.2)	59 (28.9)	
Failure	23 (1.7)	3 (1.5)	0.9
Death	125 (9.1)	25 (12.3)	0.09
Default	109 (8.0)	23 (11.3)	0.06
Transfer-out	33 (2.4)	8 (3.9)	0.14
Total	1373	204	
**Extrapulmonary TB**			0.05
Successful	42 (48.8)	1 (12.5)	
Unsuccessful	44 (51.2)	7 (87.5)	
Failure	0	0	-
Death	14 (16.3)	1 (12.5)	0.4
Default	22 (25.6)	3 (37.5)	0.08
Transfer-out	8 (9.6)	3 (37.5)	0.005
Total	83	8	
**New cases**			0.02
Successful	1044 (77.2)	139 (69.8)	
Unsuccessful	309 (22.8)	60 (30.2)	
Failure	19 (1.4)	3 (1.5)	0.8
Death	126 (9.3)	24 (12.1)	0.13
Default	126 (9.3)	23 (11.6)	0.2
Transfer-out	38 (2.8)	10 (5.0)	0.06
Total	1353	199	
**Retreatment cases**			0.06
Successful	78 (75.7)	7 (53.8)	
Unsuccessful	25 (24.3)	6 (46.2)	
Failure	4 (3.9)	0	0.6
Death	13 (12.6)	2 (15.4)	0.5
Default	5 (4.9)	3 (23.1)	0.01
Transfer-out	3 (2.9)	1 (7.7)	0.3
Total	103	13	

TB = tuberculosis.

Only 929 (94.4%) of the smear-positive TB patients had a sputum smear microscopy done after the intensive phase of treatment i.e. 853 non-elderly and 76 elderly. Of the elderly patients, 18/76 (23.7%) failed to smear convert after the intensive phase of treatment compared to 169/853 (19.8%) of the non-elderly patients (*p* = 0.06).

The treatment outcomes of elderly and non-elderly TB patients stratified by their demographic and clinical characteristics are as shown in Table 3. Across patients' residence, health facility where care was provided, treatment category and type of TB (Table 3), the rates of unsuccessful outcomes were significantly: higher among elderly compared to non-elderly patients residing in a rural area (32.1% vs 23%; *p* = 0.01), higher in elderly patients who received care at the private health facility (27.9% vs 18.9%; *p* = 0.005), higher in elderly new patients (30.2% vs 22.8%; *p* = 0.02), and higher in elderly patients with pulmonary TB (28.9% vs 21.2%; *p* = 0.01). Irrespective of HIV status, rates of unsuccessful outcomes were significantly higher in elderly than non-elderly patients (for HIV-positive cases 60% vs 33%; *p*<0.001 and for HIV-negative cases, 28.9% vs 20%; *p* = 0.005). In patients who received the longer (8-month) anti-TB regimen there was no difference in the rates of unsuccessful outcomes between elderly and non-elderly patients (*p* = 0.52). However, among those who received the 6-month anti-TB regimen rates of unsuccessful outcomes were higher in elderly compared to non-elderly patients (35.6% vs 20.6%; *p* = 0.001) (Table 3). Also, elderly patients who received the current 6-month regimen had a higher rate of unsuccessful outcomes compared to those who received the longer 8-month regimen, but the difference was not significant (35.6% vs 27.9%; *p* = 0.2).

**Table 3:** A comparison of treatment outcomes between elderly and non-elderly TB patients stratified by demographic and clinical characteristics, Nigeria, 2011–2012.

Variables	Non-elderly		Elderly		*P*-value
	Successful n (%)	Unsuccessful n (%)	Successful n (%)	Unsuccessful n (%)	
Gender					
Female	498 (79.2)	131 (20.8)	53 (69.7)	23 (30.3)	0.06
Male	624 (75.5)	203 (24.5)	93 (68.4)	43 (31.6)	0.08
Residence					
Rural	779 (77.0)	233 (23.0)	114 (67.9)	54 (32.1)	0.01
Urban	343 (77.3)	101 (22.7)	32 (72.7)	12 (27.3)	0.50
Facility					
Private	982 (81.1)	229 (18.9)	129 (72.1)	50 (27.9)	0.005
Public	140 (57.1)	105 (42.9)	17 (51.5)	16 (48.5)	0.54
Treatment category					
New	1044 (77.2)	309 (22.8)	139 (69.8)	60 (30.2)	0.024
Retreatment	78 (75.7)	25 (24.3)	7 (53.8)	6 (46.2)	0.093
Type of TB					
Extrapulmonary TB	42 (48.8)	44 (51.2)	1 (12.5)	7 (87.5)	0.05
Pulmonary TB	1080 (78.8)	290 (21.2)	145 (71.1)	59 (28.9)	0.01
HIV status					
HIV – negative	903 (80.0)	226 (20.0)	140 (71.1)	57 (28.9)	0.005
HIV – positive	219 (67.0)	108 (33.0)	6 (40.0)	9 (60.0)	<0.001
Treatment regimen					
Regimen 1	567 (74.9)	190 (25.1)	88 (72.1)	34 (27.9)	0.52
Regimen 2	555 (79.4)	144 (20.6)	58 (64.4)	32 (35.6)	0.001

Regimen 1 = 8-month regimen; Regimen 2 = 6-month regimen; TB = tuberculosis; HIV = human immunodeficiency virus.

### Risk factors for unsuccessful outcomes among elderly patients

Univariate and multivariable logistic regression analysis was performed to determine socio-demographic and clinical risk factors for unsuccessful outcomes among elderly patients (Table 4). The independent predictors for unsuccessful outcomes among elderly patients were: extrapulmonary TB case (adjusted odds ratio (aOR) 10.9; 95% confidence interval (CI) 1.1–108), and HIV co-infection (aOR 3.6; CI 1.1–11.7).

**Table 4:** Multivariable logistic regression analysis of factors associated unsuccessful treatment outcomes among elderly TB patients, Nigeria, 2011–2012.

Variables	N = 212 n (%)	Unsuccessful outcomes n (%)	Crude OR (95% CI)	Adjusted OR (95% CI)	Adjusted *p*-value
Total	212	66 (31.1)			
Older Age (years)	212	66 (31.1)	1.0 (0.99–1.1)	1.0 (0.99–1.1)	0.21
Gender					
Female	76	23 (30.3)	1	1	
Male	136	43 (31.6)	1.1 (0.6–2.0)	1.2 (0.6–2.3)	0.64
Residence					
Rural	168	54 (32.1)	1.3 (0.6–2.6)	1.6 (0.7–3.8)	0.26
Urban	44	12 (27.3)	1	1	
Facility					
Private	179	50 (27.9)	1	1	
Public	33	16 (48.5)	2.4 (1.2–5.2)	1.6 (0.6–4.2)	0.32
Type of TB					
Pulmonary	204	59 (28.9)	1	1	
Extrapulmonary	8	7 (87.5)	17 (2.0–142)	10.9 (1.1–108)	0.04
Treatment category					
New	199	60 (30.2)	1	1	
Retreatment	13	6 (46.2)	2.0 (0.6–6.2)	2.1 (0.7–6.8)	0.21
Regimen					
Regimen 2	90	32 (35.6)	1.4 (0.80–2.6)	1.4 (0.7–1.8)	0.34
Regimen 1	122	34 (27.9)	1	1	
HIV status					
Negative	197	57 (28.9)	1	1	
Positive	15	9 (60)	3.7 (1.3–10.8)	3.6 (1.1–11.7)	0.03

Regimen 1 = 8-month regimen; Regimen 2 = 6-month regimen; TB = tuberculosis; HIV = human immunodeficiency virus.

## Discussion

TB in the elderly is emerging as a major global health issue globally and the control of TB in this group is crucial for a successful TB control programme [1], [7]. This study is among few that evaluated the profile and outcomes of TB in the elderly in a high TB burden setting of Africa. We found that elderly TB cases accounted for 12.7% of all TB patients seen during the study period, and elderly TB patients had a higher proportion of men compared to women. Compared with non-elderly patients, elderly patients were less likely to be HIV-infected, and had lower rates of smear-positive TB. Also, elderly patients generally had a higher frequency of unsuccessful treatment outcomes, specifically death and treatment default, and elderly smear positive TB patients were less likely to have negative smear-conversion after the intensive phase of treatment.

The proportion of TB occurring in elderly patients in this study (12.7%) is comparable to the findings of studies in Senegal where the prevalence was 12.8% [19], as well as in India where the rates ranged between 14–16.6% of all TB cases [16]–[17]. But, our findings did not agree with results from Germany and a tertiary setting in India where TB in the elderly accounted for 36% and 4.2% of TB cases respectively [15], [24]. The reason for these differences is not clear. One plausible reason is that compared with Nigeria, the decreasing birth rate and higher life expectancy in Germany means that the aging population has a higher risk of developing TB [15]. With the increasing life expectancy in Africa [9], our finding suggests that TB in the elderly is a major issue that needs to be tackled in order to have a successful TB control programme. Consistent with earlier findings [4], [14], [16], [19], we found that elderly men had a higher rate of TB compared with women; this highlights the increased risk of the disease in elderly men in Nigeria. This finding follows the epidemiologic trend of TB in Nigeria [21]. The reasons for these higher rates in men have been suggested to be due to lower risk of TB infection in women due to biochemical mechanisms, lower socioeconomic barriers in accessing health services and higher risk of exposure to TB through social interactions in men [25]–[26].

Compared with non-elderly patients, elderly individuals were less likely to be HIV-infected in this study. This agrees with a previous study [14]; but the reason(s) for this difference are not clear. It probably reflects that epidemiologic differences exist in HIV infection rates between elderly and non-elderly adults as well as its overall impact on the burden of TB between them [27]. Population-based data of HIV infection rates reported by the United Nations generally report only prevalence rates for individuals who are 49 years old or below [28]. To better reflect the longer survival of HIV-infected persons and the ageing of the HIV-infected population in the antiretroviral therapy era, indicators of the prevalence of HIV infection should be expanded to include people older than 49 years of age. Extrapolation of prevalence data of the younger population suggests that the rate of HIV-infection in older individuals (>49 years) was lower compared to younger ones in various regions including Nigeria [29]. These differences indicate that the profile and burden of TB/HIV co-infection in the elderly warrant further investigation.

Consistent with findings from Germany, India, Mexico and Taiwan, we have shown that smear-positive microscopy results were seen less frequently in the elderly [3], [11], [15], [17], [24]. These recent findings contradicts the conclusion of a meta-analysis done almost two decades ago which did not find differences between elderly and non-elderly adult TB patients regarding rates of positive acid-fast bacilli in their sputum [5]. Our finding of lower smear positivity rates in the elderly patients may be because of difficulties in obtaining adequate sputum from them due to their inability to produce sputum, or by producing lower quality sputum containing mainly saliva [11]. Our study suggests the need to incorporate other diagnostic techniques like bronchoscopy, sputum culture and Xpert MTB/RIF while evaluating elderly patients for TB. This should go hand-in-hand with other simple interventions such as sputum-submission instructions on how to expectorate sputum which have been shown to improve smear-positivity of pulmonary TB cases [30].

The negative smear conversion of smear positive TB cases is an important determinant of treatment success [31]. This study showed that elderly smear positive TB patients had lower smear conversion rates at the end of intensive phase of treatment. This is similar to previous studies in India and elsewhere in which the sputum conversion rates at the end of the intensive phase were found to be lower in the elderly in comparison to the non-elderly TB patients [7], [17], [24]. The possible reasons for this may include advanced disease in the elderly with higher bacillary load resulting in delayed clearance of the bacilli, higher rate of adverse effects of standard anti-TB medications in the elderly leading to dose reduction, anti-TB dose reductions due to co-morbid conditions such as renal failure, and reduced absorption of the anti-TB medications in the elderly [7], [17], [24], [32].

Treatment success rate were lower and unsuccessful treatment outcomes, mainly death and default, were more frequent in the elderly than non-elderly patients in this study. This supports the finding of other studies in Africa, Asia, North America, and Europe [4], [10]–[12], [14]–[17], [19]. The higher deaths observed among elderly TB patients may be due to increased levels of co-morbid conditions and factors like diabetes mellitus, hypertension, excess alcohol use, smoking and cardiovascular diseases in this age-group as well as higher proportion of patients with advanced disease in the elderly [16], [19], [33]. Given that this is a retrospective review of TB registers, we could not assess the extent of these problems and their impact on treatment outcomes. Assessment and recording of co-morbidities like diabetes mellitus should be included routinely in the management of TB as this will ensure early management of this co-morbidity with TB. The higher rates of treatment default in the elderly may be due to poor memory of appointment dates, higher adverse drug reactions leading to poorer adherence to treatment, co-morbid diseases and inability to visit the treatment centres due to absence of a family member that accompanies them to the treatment centre [16], [19]. Given that more than four fifths of the elderly patients in this study resided in the rural area, treatment default in the elderly may be lowered by providing quality geriatric health services at the primary care level to address the varying needs of the elderly patients in rural settings of Nigeria.

This study showed that treatment success rates of elderly were inferior to that of non-elderly patients who were rural residents, had extrapulmonary TB, treated in a private facility, received the current 6-month anti-TB regimen as well as those with or without HIV infection. In order to better understand these differences in treatment success rates, we assessed the determinants of unsuccessful outcomes in elderly patients. Elderly patients who received the 6-month regimen had higher proportions of unsuccessful outcomes than those given the 8-month regimen. Although the crude and adjusted odds ratios did not reach statistical significance, this study suggests the need to further evaluate the effect of adjusting the duration of the current 6-month anti-TB regimen on treatment outcomes among elderly TB patients in resource poor settings. HIV co-infection and presence of extrapulmonary TB were independent predictors for unsuccessful outcomes in the elderly – these factors are not specific to the elderly patients alone [34]. Such individuals at risk of unsuccessful outcomes need to be identified early and closely monitored to ensure a successful treatment outcome.

There are several limitations to this study. The study was based on routine surveillance data. We were not able to assess for relationships between TB treatment outcomes in the elderly and other important variables like diabetes mellitus, renal failure, and other co-morbidities. Also, as TB/HIV collaborative activities were recently expanded in Ebonyi state, data on HIV status of all patients were available; but data on those who were started on antiretroviral therapy were not adequately captured in the tuberculosis treatment registers. Thus, other data like details of antiretroviral therapy use, cotrimoxazole prophylaxis and CD4+ count were not available for analysis. Furthermore, there were no information on the duration of symptoms before treatment, chest radiography, toxicity of anti-TB drugs, or causes of death; these variables may affect treatment outcomes.

In conclusion, this study has shown that TB in the elderly occurred more frequently in men; they are less likely to have smear positive disease, and less likely to show negative smear conversion after the intensive phase of treatment. Also, compared with non-elderly TB cases, elderly TB patients are more likely to have unsuccessful treatment outcomes. The findings of this study have a number of practice and policy implications. We recommend that: 1) special interventions to reduce treatment default and mortality in elderly patients with TB needs to be implemented; 2) specific strategies to reduce unsuccessful outcomes among elderly patients in the identified high-risk groups should be implemented; 3) expansion of case finding and treatment of HIV among elderly TB patients should be improved; 4) Further studies on the impact of the current 6-month regimen on TB treatment outcomes in the elderly needs to be carried out and its relationships with the duration of treatment assessed; and 5) TB treatment registers in Nigeria and elsewhere should be modified to collect additional data such as co-morbidities, adverse drug reactions, and concomitant therapies from TB patients treated.

## Supporting Information

Table S1 **Dataset on profile and treatment outcomes of tuberculosis in the elderly in Southeastern Nigeria, 2011–2012.**
(MDB)
